# A new era in identification of tick genera; artificial intelligence for precision and speed

**DOI:** 10.7717/peerj-cs.3291

**Published:** 2026-01-27

**Authors:** Ibrahim A. Ame, Abdullahi Ibrahim Umar, Cenk S. Ozverel, Erdal Şanlıdağ, Ayse Seyer, Fadi Al-Turjman, Tamer Sanlidag

**Affiliations:** 1 Artificial Intelligence, Software, and Information Systems Engineering Departments, Research Center for AI and IoT, AI and Robotics Institute, Near East University, Nicosia, Cyprus; 2 Department of Biomedical Engineering, Near East University, Nicosia, Cyprus; 3 DESAM Research Institute, Near East University, Nicosia, Cyprus

**Keywords:** Hyalomma, Rhipicephalus, Prediction, Tick, Borne disease, Deep learning, CNN, Tick

## Abstract

**Background:**

The occurrence of pandemics in the last 20 years highlighted the unpreparedness of healthcare systems. There is a worldwide increased trend in the vector borne diseases. Ticks are one of the most common organisms that play a vital role in global ecosystem as well as being vectors of diseases affecting human and livestock. They are able to carry infectious agents that might cause illnesses including paralysis and to some certain extend death. Therefore, it is crucial to identify different genera of ticks to track infectious agents. Conventionally, tick classification is done by acarologists who are experts in the field. For this reason, the identification process is carried out in a difficult and time-consuming manner.

**Method:**

The aim of the study was to develop a web-based application by using artificial intelligence-based algorithms to easily identify Hyalomma and Rhipicephalus ticks, which are the most abundant genera in Northern Cyprus, with high sensitivity and accuracy. The experimental procedure is structured based on five phases. Phase 1 revolves around data collection in which pictures of 35 identified ticks are taken by experienced acarologists and the curation of non-tick images (spiders, beetles, mites, mosquitos and scorpions). Phase 2 revolves around pre-processing steps and data split. Phase 3 involves training and testing custom Convolutional Neural Network (CNN), Visual Geometry Group 16 (VGG16), Residual Network 50 (ResNet-50) using 6,972 images (3,486 images for each class) for discrimination between ticks and non-ticks and 9,556 images (4,778 images for each class) for the discrimination between Hyalomma and Rhipicephalus. Phase 4 revolves around performance evaluation. Phase 5 is characterized by development of a web-based application (I-TickNet), created to enable a widespread use of the tick classifier.

**Results:**

The performance evaluation and comparison of the model performance has shown that ResNet50 achieved the best result for binary classification of tick and non-tick (experiment A) with accuracy of 100% and Area Under the Curve (AUC) score of 100%. Moreover, VGG16 achieved the best result for binary classification of ticks (experiment B) with an accuracy of 96.97% and AUC score of 99.55% respectively. All the three models were employed for the development of artificial intelligence/Internet of Things (AI/IoT) framework known as I-TickNet for real-time and on-spot classification of tick images. In conclusion, this study provided a web-based application that can identify two distinct tick genera with high accuracy and sensitivity. The application developed enabled a user-friendly interface to identify genera without requiring any expertise.

## Introduction

In past 20 years, the occurrence of outbreaks has increased. The recent COVID-19 pandemic has indicated the unpreparedness of healthcare systems. Vector borne diseases are also frequently seen in geographic regions that were never present before. This might be attributed to various causes including climate change, wars, immigration crises and economic crises around the world. Ticks are among the vectors that possess approximately 900 distinct species. In addition to their diverse life cycles, they possess distinct morphological and physiological characteristics. They are capable of consuming more than 100 times their body mass in blood and can feed for extended periods. Ticks are regarded as the second most common carriers of infectious diseases to humans after mosquitoes and are known vectors of some of the most dangerous infectious agents including Crimean Congo Hemorrhagic Fever Virus and Rickettsia. Considering the fact that the risk of tick-borne diseases depends on the tick species, therefore, tick identification is crucial for preventing infection (Barker & Murrell, 2004; Estrada-Peña, 2015; Dantas-Torres, 2018; Shah et al., 2023).

Identification of ticks’ species relies on standard taxonomic approaches which require skilled expert in the field of entomology and it is time consuming. Classification of ticks using morphological techniques is hindered by two main challenges which include high inter-genera similarity (where different tick genera share morphological similarities) and high intra-genera variability (where ticks from the same species differs) (Ruenchit, 2021; Akbarian et al., 2021; Justen et al., 2021; Xu et al., 2021). Moreover, identification and classification of ticks without specializing in entomology is highly difficult due to similarities in genera appearances and small size of ticks (Justen et al., 2021; Luo et al., 2022; Vieira Lista et al., 2022).

Scientists are currently engaging the use of Artificial Intelligence (AI) in the health and life sciences. These multidisciplinary studies might employ computer-assisted technology which comprises of image processing, image detection, segmentation, recognition and classification using machine learning (ML) and deep learning (DL) architectures which can be further classified into Artificial Neural Networks (ANNs) and Convolutional Neural Networks (CNNs) (Irkham et al., 2022; Ibrahim et al., 2023).

DL-based models are capable of identifying features and patterns from training set in order to accurately predict or classify new data. DL-based models have display excellent result in solving computer vision tasks such as image identification, recognition, classification *etc*. (Tamuly, Jyotsna & Amudha, 2020; Wang, Fan & Wang, 2021). However, training customize built CNN model from scratch has some drawbacks which include the requirement of training using a substantial amount of data in order to achieve significant performance (Salehi et al., 2023). One of the popular techniques to address these shortcomings revolves around the use of pre-trained models (*i.e*., transferability or transfer learning) where knowledge is transferred from networks trained using larger dataset such as the ImageNet dataset (*i.e*., known source or already trained task) to new task. Transfer Learning (TL)-based models are predominantly used in medical applications due to its numerous benefits over customized models. Some of the benefits of the TL-based approach include high accuracy or efficiency, time and cost saving (Zhuang et al., 2020; Ibrahim et al., 2024; Kim et al., 2022). There are several pre-trained models that are available for computer vision applications. The most common pre-trained models include Residual Networks (ResNets) (ResNet-50, ResNet-101 *etc*.), VGGNets (VGG16 and VGG19), AlexNet, Inception, Xception, DenseNet, EfficientNet *etc*.

As aforementioned, detection or identification of tick species is crucial for surveillance studies to track the possible occurrence rate of an outbreak (Omodior et al., 2021). In the present century, whole world is witnessing the rise of artificial intelligence/Internet of Things (AI/IoT)-enabled frameworks that allow access to users from all over the world to upload medical images for on-spot or real-time identification. Several IoT-based platforms that are embedded with DL models have been developed for the detection of COVID-19 from X-ray and Computed Tomography (CT) scan (Irkham et al., 2022), skin cancer from dermoscopic images, brain cancer from Magnetic Resonance Imaging (MRI) images (Ibrahim et al., 2025a, 2025b), breast cancer from histopathological images *etc*.

### Motivation

Ticks across various region of the world serve as vectors for numerous pathogens such as bacteria, viruses, protozoa *etc*. Different species of ticks serve as reservoirs of bacteria which can be transmitted transstadially and transovarially. Among different types of species, the most common species found in Cyprus includes *Rhipicephalus and Hyalomma* (Tsatsaris et al., 2016). Accurate discrimination of these tick species is crucial for public health, veterinary medicine, and ecological research as they help in risk disease assessment, proper surveillance and emerging threats, public awareness and prevention. Manual classification of tick species is prone to miss-classification or miss-identification and time consuming (Justen et al., 2021; Akbarian et al., 2021). Therefore, there is need for developing automated system that can distinguish between different species.

Despite plethora of studies in the literature on the application of DL or computer-aided detection (CAD) for image classifications, only few studies have attempted to discriminate between Hyalomma and Rhipicephalus. Moreover, majority of the studies only implemented single model, pre-trained models and limited number of datasets. Moreover, there is scarcity of AI/IoT-powered platform for real-time classification of tick species and non-tick images. Consequently, majority of studies only reported models implemented in a computer, which is not freely accessible by patients and healthcare experts. In order to address these issues, this study proposed a framework known as I-TickNet, an AI/IoT-powered platform designed using custom shallow CNN (6-layer), and pre-trained models (Visual Geometry Group 16 (VGG16) and Residual Network 50 (ResNet-50)) for accurate discrimination of two tick species and the subsequent deployment of the models on to a web app for real-time detection. Both customized model and pre-trained models are deployed in order to established the best performing model.

### Literature survey

The study by Luo et al. (2022) applied AI-based techniques to classify ticks. The study acquired image dataset from a tick passive surveillance program of the most encountered human-biting ticks, which include *Dermacentor variabilis, Ixodes scapularis and Amblyomma americanum*. The dataset comprises of 12,000 high-resolution micrographs molecularly confirmed by a genera-specific TaqMan polymerase chain reaction (PCR) assay. The dataset is split into 90% for training (10,800) and validation and 10% for testing (1,200). In order to increase the diversity of training set, data augmentation techniques were implemented *via* zooming and random rotation. The images are trained and tested using five pre-trained CNN, which include MobileNet V2, Inception v3, DenseNet121, ResNet50 and VGG16. The evaluation of the models and comparative analysis has shown that the Inception V3 model achieved the best result in terms of accuracy with 99.5%.

Xu et al. (2021) developed smartphone app known as TickPhone app for rapid tick identification of three types, which include *Amblyomma americanum* (line star tick), *Ixodes scapularis* (deer tick) and *Dermacentor variabilis* (dog tick). The experimental procedure is set up based on several steps which include collection of image data, optimization of DL models, training and testing and development of smartphone app. The study acquired dataset from the Connecticut Veterinary Medical Diagnostic Laboratory and photos taken using smartphones. In order to enlarge that dataset, the study conducted data augmentation *via* flipping, shifting, zooming, shearing and rotation which resulted in more than 2,000 images. In order to identify ticks, modified and optimized LeNet is used to extract features followed by classification using Support Vector Machine (SVM). The optimized DL model achieved 85% validation accuracy. Moreover, the study developed TickPhone app and the testing of the app has shown that it can identify 31 tick genera resulting in 95.69% accuracy.

In the study reported by Omodior et al. (2021), the authors proposed the use of CNN for the detection of ticks. The overall methodology revolves around data curation, development of customize CNN, implementation of CNN and pre-trained networks and performance evaluation. The study developed a dataset which comprises 2,130 images of four tick genera, which include *Haemaphysalis sp, Dermacentor variabilis, Ixodes scapularis* and *Amblyomma americanum*. The images are further partition into three subset which include 70% for training 20% for validation and 10% for testing. The images are trained and tested using shallow custom-built CNN and pre-trained ResNet50. The comparison between shallow custom built CNN and pre-trained ResNet50 has shown that customized CNN achieved greater accuracy of 80% using subset of unseen dataset.

The development of Inception V3 (TickIDNet) which is DL-based approach for the ternary classification of ticks is reported by Justen et al. (2021). The first step taken in order to achieve accurate classification revolves around the generation of image dataset which comprises of 12,177 images of three common ticks found in the US (*Ixodes scapularis, Dermacentor variabilis and Amblyomma americanum*). The images are subsequently processed based on quality and 1,273 are discarded while 10,900 images are kept. The processed images are further split into 70% for training (7,625), 20% for validation (2,181) and 10% for testing (1,094). In order to maximize the dataset, several data augmentation techniques were adopted which include rotation, cropping, and flipping which led to the generation of 95, 119 images from the initial 7,625 images. Evaluation of the TickIDNet on two testing set resulted in 87.8% accuracy, 87.73% weighted F1-score, and 0.7895 Kappa agreement score am using a user generated test set and also 91.67% accuracy, 91.55% weighted F1-score, and 0.875 Kappa agreement score using a laboratory test set (300 images with 100 images per each class).

The real-time automated detection or identification of tick genera (binary classification between black-legged (*Ixodes scapularis*) and over six other non-black-legged genera) using computer vision technique based on CNN is reported by Akbarian et al. (2021). The research is designed based on several steps, including image data collection, pre-processing, training and testing. The first step involves data collection from Public Health Ontario, which comprises of 6,294 images of black-legged (*Ixodes scapularis*) (41%) and over six other non-black-legged genera (59%). Moreover, the images are split into training and testing. The training set undergoes data augmentation *via* zooming, flipping and random rotation. The acquired images were trained and tested using 7-layer customized CNN and Inception-ResNet CNN. The last step includes the development of web for real-time identification. Evaluations d comparison between untrained and pre-trained model has shown that InceptionResNet achieved 92.04% accuracy.

The summary of the related work is presented in Table 1.

**Table 1:** Summary of related work.

References	Model	Number of images	Accuracy
Luo et al. (2022)	Inception V3	12,000	99.5%
Xu et al. (2021)	LeNet-SVM	–	95.69%
Omodior et al. (2021)	ResNet50	2,130	80.00%
Justen et al. (2021)	TickIDNet	12,177	91.67%
Akbarian et al. (2021)	InceptionResNet	6,294	92.04%

## Materials and Methods

This study follows a pipeline that comprises of five steps, which involve curation of image dataset, image processing, model development and implementation of pre-trained networks, performance evaluation and web development for accurate, fast and real-time detection of ticks. The data comprises of images of ticks taken under a microscope and non-tick images (spiders, beetles, mites, mosquitos and scorpions). The dataset was split into a ratio of 60:30:10.

CNN models were used to classify these images into the correct group because they offer the best prediction when using images. These CNN methods were proven to be effective at classifying different types of items from one another, their strong feature extraction properties were crucial in identifying the subtle differences between ticks and non-ticks as well as *Hyalomma and Rhipicephalus* species. The selection of CNN for this study was based on its proven effectiveness on distinguishing the differences between images. In this study we discriminate between two ticks that had very subtle differences. Traditional AI algorithms like SVM were considered but not selected because we had to manually engineer our own feature extraction algorithm. This would prove very difficult and almost impossible as the ticks had details that even experts could sometimes misclassify. A preliminary test was conducted using a small subset of the dataset to evaluate their accuracy, speed, and generalization ability. We chose to use CNN models like pre-trained ResNet50 and VGG16 due to their efficiency and their ability to detect underlying patterns in images. Additionally, ResNet50 was chosen as it was a deep network that had residual connections in its architecture to mitigate vanishing gradients if some of these features were lost in the early layers.

Speed of prediction (less than 5 s) is regarded as one of the satisficing metrics for this study. The models were evaluated according to several metrices including accuracies, losses, precisions, recalls, F1-scores and Receiver Operating Characteristic (ROC) curves. This was crucial to ascertain if the models had trained properly and did not overfit the dataset. Accuracy was important as it stated how probable a model would be able to predict the correct result. The loss of the model was a measure to see how well the model trained and if it had gotten close to its global minima with gradient descent. With Precision, an evaluation was required to see how many images the model could get correct in the testing phase, this would be a lens to see how the model would perform on images it had not seen before. This is different from recall, as it checks the actual positive cases that were correctly identified by the model. The F1-score was used to check if the recall and precision were correct in order to ensure that the models can confidently discriminate between Hyalomma and Rhipicephalus. Finally the ROC curves were required to ensure that we were getting close to a perfect classifier.

### Code repository

Access to a public repo containing all the links for all the dataset and code used in this study can be found at https://github.com/ameibrahim/ITICK.

### Data collection and processing

A comprehensive study of Hyalomma and Rhipicephalus (*n* = 35) ticks’ genera were participated by an experienced acarologist. 5–7 adult ticks were collected from six different dogs located in Northern Cyprus. The brand of the microscope use is Bresser GmbH (Germany) Biolux Touch (digital LCD microscope) with 4×, 10×, and 40× magnifications. It has integrated digital camera allows users to take photos and record videos directly onto a memory card. Idiosomic (body) ventral and dorsal views and capitulum (head) ventral and dorsal views including the palps and hypostome were all captured.

As a result of this identification process, images were taken from different angles for each tick sample, leading to a total of 9,556 images (4,778) for each class, in order to ensure the two classes are balanced. While for the discrimination of tick and non-tick, 6,972 images are used. Both Hyalomma and Rhipicephalus are grouped in one class (*i.e*., tick with 3,486 images). While non-ticks comprise of a combination of arachnids and other insects, spiders, beetles, mites, mosquitos and scorpions (3,486 total images).

**Figure 1:**
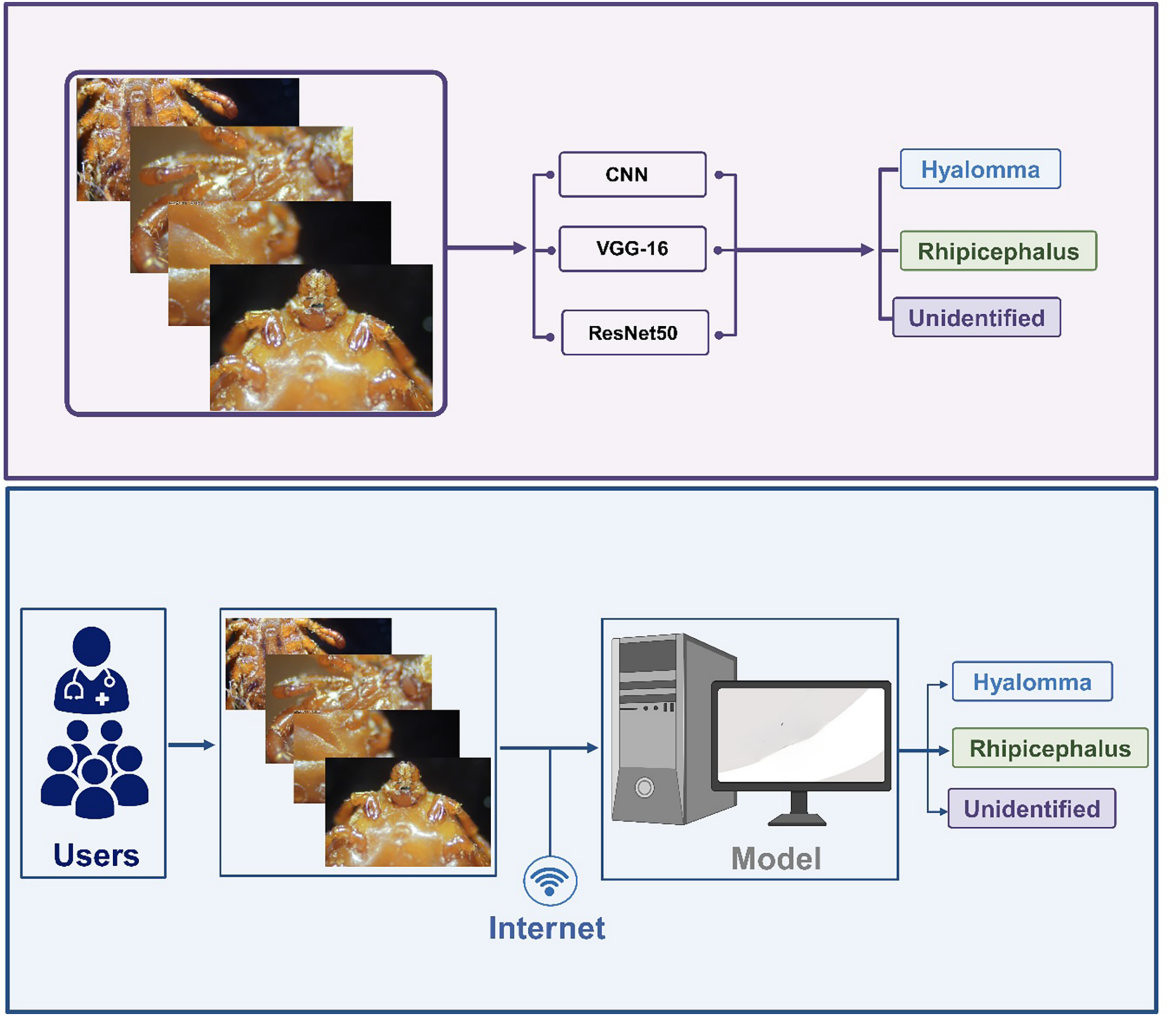
Overall methodology.

The dataset undergoes several processing steps which include labelling, cleaning, restructuring *etc*. The process images are further trained and tested using a customized shallow 5-layer CNN, VGG-16 and ResNet-50. The next step revolves around performance evaluation based on accuracy. The best performing model is selected for the development of IoT-based framework for accurate and real-time classification of ticks. The overall methodology is summarized in Fig. 1.

### Description of dataset

**Figure 2:**
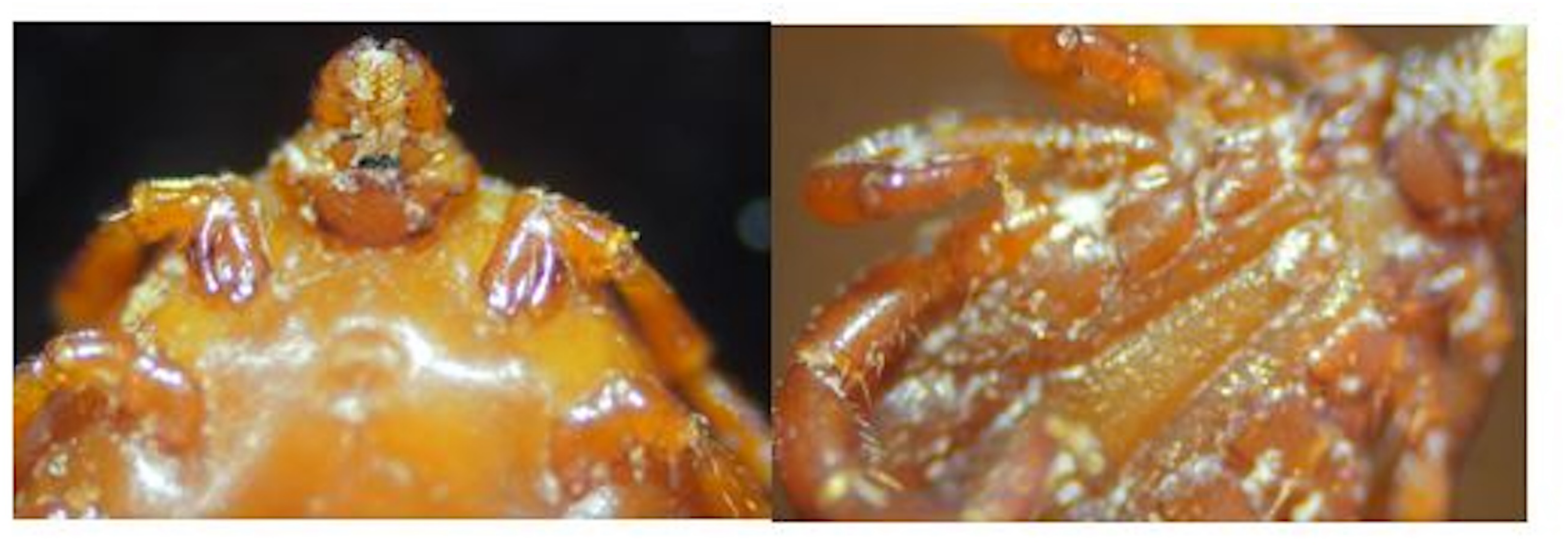
A microscopic view of ticks.

Training ML and DL models with sufficient data is crucial for achieving high performance. The two ticks genera used in this study vary from one another. Hyalomma are characterized by their long mouthpart (*i.e*., longirostrate) and legs with pale rings. In terms of texture, there scutum or conscutum are colored brown. Rhipicephalus on the other hand are characterized by their hexagonal shape. In terms of texture, they vary from yellowish-brown to reddish-brown, with a dark inornate brown scutum (Geevarghese & Mishra, 2011) as shown in Fig. 2.

### Data split and data augmentation

Partitioning of dataset for ML and DL application may vary due to several factors such as dataset size, complexity, and data availability. Over the last five decades, several data split ratios have been proposed, which include two class and three class split which include 60:40, 70:30, and 80:20 for training and testing as well as 60:20:20, 70:15:15 and 80:10:10 for training, validation and testing respectively. The datasets were split into a 60% ratio for training, 30% ratio for validation and 10% for testing. This yielded 4,181, 2,090 and 699 images respectively for the tick non tick dataset (*i.e*., dataset A) and 5,733, 2,866 and 958 images respectively for the tick classification (*i.e*., dataset B). Data split is presented in Table 2. During training, the images were augmented using Random Flips, Rotation, Zoom, Translation, Contrast and Brightness. The augmentation was carried out during training over 35 epochs (*i.e*., 4,778 newly augmented images are generated for every epoch).

**Table 2:** Data split of training, validation and test sets.

Set name	Percentage	Image count
**Dataset A**		
Train	60	4,181
Validation	30	2,090
Test	10	699
**Dataset B**		
Train	60	5,733
Validation	30	2,866
Test	10	958

### Deep learning models

#### Visual Geometry Group (VGG)

VGG is one of the earliest CNN models developed in 2014 by researchers working at Oxford known as Simonyan and Zisserman for computer vision task. The DL network has gained popularity and is widely implemented in image classification due to its simplicity. The model was developed in order to address the limitations of traditional ML models such as identification of complex relationships between data. This called for the need to develop deeper network that can identify intricate features and patterns in images. The network achieved second spot in the ILSVRC competition with 92.7% top-5 test accuracy (Simonyan, Vedaldi & Zisserman, 2013; Sengupta et al., 2019).

The two popular VGG networks include VGG16 and VGG19. Other VGG networks include VGG11 and VGG13. The numbers assigned to each network represent the number of layers. The networks comprise of different operations, which include convolution and max pooling. Both VGG16 and VGG19 comprise of convolutional layers, pooling layers and fully connected layers. The convolutional layers in VGG networks are characterized by small 3 × 3 convolutional filters. The convolutional layers enable the network to capture a wide range of features at different scales. Convolutional layers are followed by pooling layers (placed after every 2 or 3 Convolutional layers). Max pooling layer is used in VGG networks in order to minimize spatial dimensions while simultaneously retaining significant features. The last part of the network is characterized by fully connected layers which merge the learned features for class prediction using SoftMax classifier. Moreover, the network utilizes ReLu activation function through the network which helps introduce non-linearity. VGGNET are trained using stochastic gradient descent (SGD) while in order to minimize overfitting, Dropout and regularization technique are used (Sengupta et al., 2019; Mascarenhas & Agarwal, 2021).

VGG16 is made of 16 layers in which 13 layers are convolutional layers and the remaining three are fully connected layers. The layers are structured into six units. The first two units are characterized by two convolutional layers using 3 × 3 filters followed by max pooling layer. The third, fourth and fifth layers are made of three convolutional layers followed by max pooling layer. The sixth unit comprises of three fully connected layer while SoftMax activation is applied to the output of the third fully connected layer for classification. The network accepts input with a size of 224 × 224 × 3 and can classify input into 1,000 categories (Mascarenhas & Agarwal, 2021).

#### ResNet-50

The concept of residual networks is introduced in order to address the issue of vanishing gradient suffered by VGG networks. After the introduction of Le-Net and AlexNet, scientists began stacking more layers to neural networks which result in poor performance and difficult to train. This problem is solved as a result of the development of ResNet by He et al. (2016). The network achieved top spot in the 2015 ILSVRC classification competition with a top-5 error rate of 3.57%. ResNet addressed the problem of vanishing gradient by the introduction of skip connection which provides an alternate shortcut path for the gradient to flow through (Targ, Almeida & Lyman, 2016). The ResNet architectures are made up of residual blocks and operations, which include convolution, pooling, padding *etc*. (Mascarenhas & Agarwal, 2021). There are several variants of ResNet with ResNet-50 and ResNet-101 as the most popular ones. Other variants include ResNet-18, ResNet-34, ResNet-152, ResNext, InceptionResNetV2 *etc*.

#### Customized CNN

Portions of this text were previously published as part of a thesis by the first author (Ibrahim, 2023).

**Figure 3:**
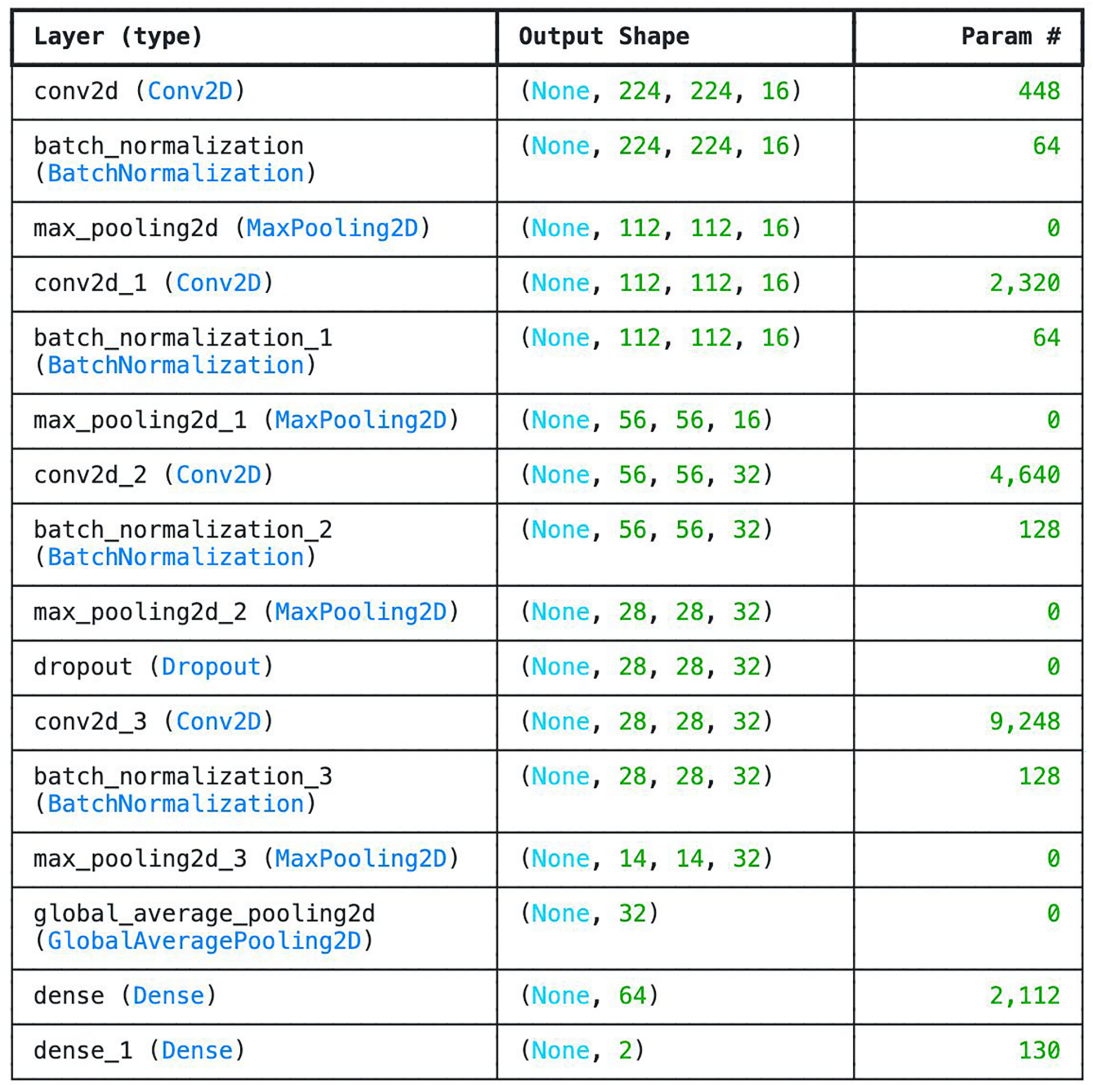
Parameter list of basic CNN architecture.

We developed six-layer CNN known as basic model. The 6 layers in the network comprise of four convolutional layers and one fully connected layer designed with SoftMax for classification. Other layers such as the average pooling layer and flattening layers were not counted. The choice of 6-layer CNN as the base model is to check the efficiency of the network in classifying tick images. Compare to other deeper architectures, this model is very shallow as it doesn’t have sufficient number of layers in order to be able to understand underlying features. However, one of the advantages of the model is that is very small (*i.e*., 4.2 MB) which can be beneficial in memory constraints systems. Moreover, the model consumes the least amount of Random Access Memory (RAM) and disk space. The parameters and the number of layers of the customized CNN are shown in Fig. 3.

### Model training

Training ML models requires having an environment that can facilitate efficient passage of data and utilizing enough memory so that the model can train quickly. Thus, all the three models implemented in this study were babysat. The models are trained based on gradient descent with the main objective revolving around finding the global minimum of the function in order to make learning more efficient and very low loss. Moreover, optimizers are used during training to make gradient descent run faster. Initially, both Stochastic Gradient Descent SGD and Adam optimizers are used interchangeably between models to establish the best approach. Evaluation of the two optimizers has shown that Adam optimizer performed faster and more efficient than SGD and therefore, it is selected as the default the optimizer. Equation (1) present Stochastic Gradient Descent and Adam Averaging Summations from Sums of the Loss Function:

(1) $$J\left( \theta \right) = \displaystyle{1 \over m}\mathop \sum \nolimits_{i = 1}^m L\; ({y_i},f\left( {{x_[},\theta } \right).$$

In order to train the models, the codes of setting up the environment and loading the data as well as training the model were split into cells and run independently within a Jupyter file. Jupyter files can handle chunks of code in a cell one at a time. Therefore, if one of the cells crashes, data from other cells can be retained. Thus, this feature helps in minimizing the time needed to run previous cells again. Moreover, the efficiency of the Jupyter kernels aid in training multiple models on a more powerful machine.

Three models are implemented in this study which includes untrained shallow CNN and two pre-trained models (VGG16 and ResNet50). The three models were trained using a server equipped with powerful resources. The models were trained using on a combination of Graphics Processing Unit (GPU) and Central Processing Unit (CPU), depending on what was free on the server, as it was training them in parallel. This helped reduce the time it took to train the models from days to just minutes. Paperspace server is used which significantly reduced the amount of training time. The server was a Linux machine running Ubuntu 22.04 with dual 16 GB GPU cores, 16 CPU cores and 96.6 GB RAM. The two cores were run separately to help train two models in parallel over two different Jupyter files under the same kernel. Several input sizes were evaluated which include 32 × 32, 64 × 64, 128 × 128 and 256 × 256 and 128 × 128 is selected as it exhibited optimum size for accuracy ratio. Moreover, the models were trained using 35 epochs. Transmit was employed to fetch the models after training to a personal workspace and subsequently tested locally over the frontend user interface.

### Web development

Web application was created to complement the models implemented in this study. Predicting images through the Command Line Interface (CLI) can be extremely cumbersome considering the fact that command must be type in order to run each prediction. Thus, using an application that can communicate with models on the backend and produce a visual look of the results generated by the model equips users to predict images of different kinds through different models. These results are automatically saved for future review and comparisons.

The web application was built using an array of languages that weld together a coherent experience for discriminating between two tick species. The concept of the application is to use an Application Programming Interface (API) to communicate with the model backend and return results to the frontend. The model was built using Python and a popular framework known as TensorFlow. It’s only fitting that the API follows suit as well. All models were saved as keras files that ensured compatibility for calling the API. The API was built with Flask, a very popular tool for building sites and APIs using Python. It was served with a Web Server Gateway Interface (WSGI) framework named Waitress over a safe port.

The API lives on a different server from the frontend user interface. API calls are produced using a URL that is passed from the front end to the backend. On a successful result, the server returns a 200 status response with a valid result. The front end picks up the results and saves all the metadata into the database. This process varies with internet speeds as the image needs to move from the frontend server to the API server. The larger the image uploaded, the longer it will take. Therefore, the use of 128 × 128 input sizes made the whole process significantly smoother, and the results came back very quickly.

## Results

In this study, we implemented both untrained (shallow CNN developed from scratch) and pre-trained models (ResNet50 and VGG16) for the accurate classification of two ticks genera and unidentified cases. The models’ performances are evaluated using several metrics which include accuracy, precision, recall, sensitivity, specificity, F1-score and AUC. Mathematically representations of the metrics are outlined below:

(2) $${\rm Accuracy}= \displaystyle{{TP + TN} \over {FP + FN}}$$

where TP: True positive TN: True negative FP: False Positive and FN: False Negative.

(3) $${\rm Precision}= \displaystyle{{TN} \over {TP + FP}}$$

(4) $${\rm Recall}= \displaystyle{{TP} \over {TP + FN}}$$

(5) $${\rm Sensitivity}= \displaystyle{{TP} \over {TP + FN}}$$

(6) $${\rm Specificity}= \displaystyle{{TN} \over {FP + TN}}$$

(7) $${\rm F1{\mathrm{-}}score}= 2\times \displaystyle{{Recall\times Precision} \over {Recall + Precision}}.$$

### Performance evaluation

#### Tick *vs* non-ticks

**Figure 4:**
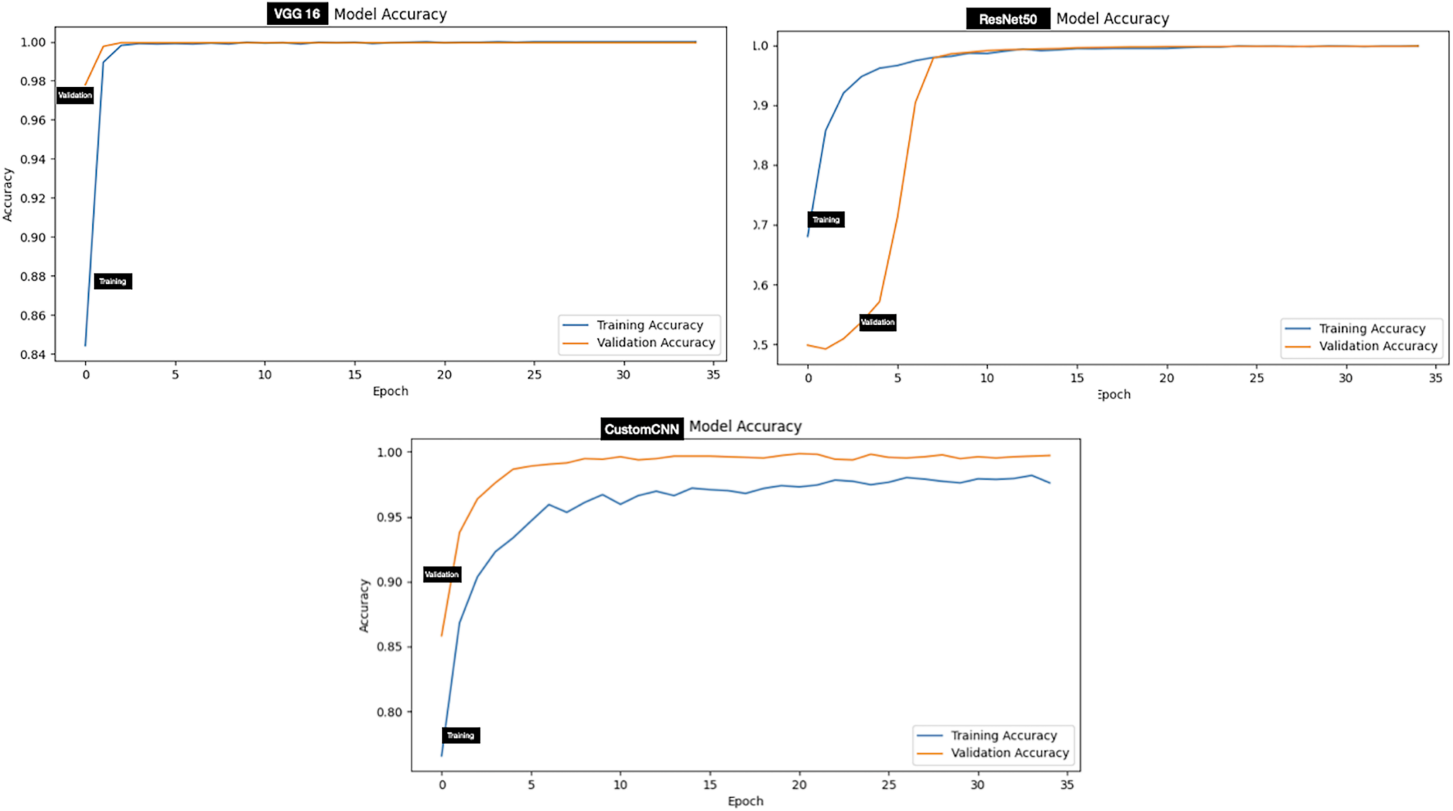
Accuracy graphs of ticks *vs* non ticks.

The performance evaluation of models (customized CNN, ResNet50 and VGG16) trained and tested using dataset A (tick *vs* non-tick) produced excellent result across all metrics. Assessment of the models on the testing set has shown that ResNet50 attained the highest score across all metrics, resulting in 100%, accuracy, precision, Recall, F1-score, Specificity, AUC, Mathews Correlation Coefficient (MCC), Cohen’s Kappa (CK), balanced Accuracy and Jaccard Index (IoU). While both VGG16 and custom CNN achieved similar result across all metrics as summarized in Table 3. The testing accuracy graphs of the three models are presented in Fig. 4.

**Table 3:** Performance evaluation of customized and pre-trained models.

Models	Round	Acc (%)	Prc (%)	Rec (%)	F1-S (%)	Spe (%)	AUC (%)	MCC (%)	CK (%)
**Dataset A (Tick *vs* non-Tick)**									
VGG16	4	99.86	99.71	100	99.86	99.71	100	99.71	99.71
ResNet50	1	100	100	100	100	100	100	100	100
CNN	5	99.86	99.71	100	99.86	99.71	100	99.71	99.71
**Dataset B (Tick identification)**									
VGG16	3	96.97	96.11	97.91	97.00	96.03	99.55	93.96	93.95
ResNet50	4	94.89	93.00	97.08	94.99	92.69	99.01	89.86	89.77
CNN	5	93.35	96.47	91.23	93.78	96.66	97.82	88.02	87.89

**Note:**

Acc, Accuracy; Prc, precision; Rec, recall; F1-S, F1-score; Sen, sensitivity; Spe, specificity; CK, Cohen’s Kappa.

#### Tick identification (Hyalomma and Rhipicephalus)

**Figure 5:**
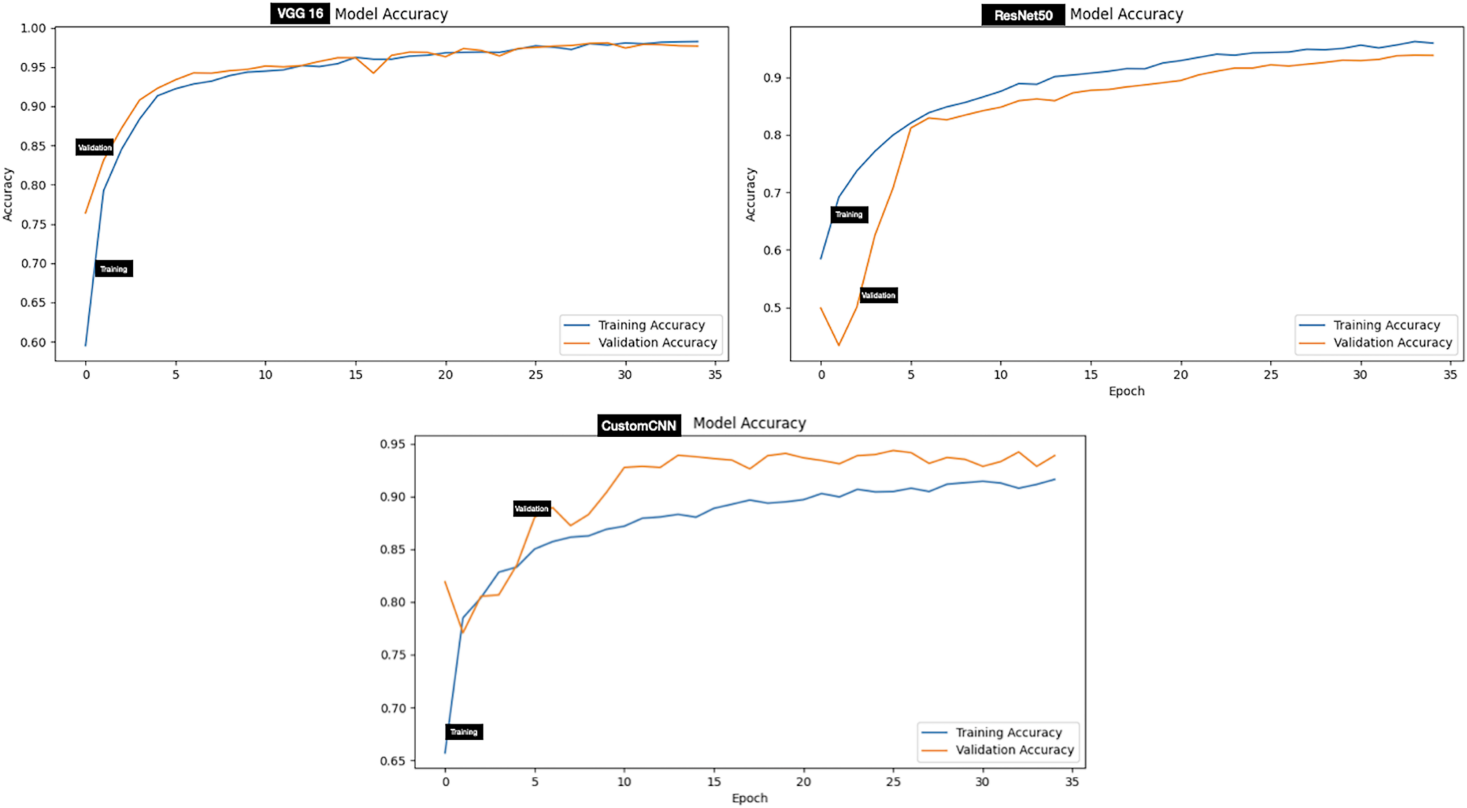
Accuracy graphs of ticks identification.

Evaluation of the models on test set of dataset B (two classes which include Hyalomma and Rhipicephalus) produced good result across all metrics. Contrary to dataset A, VGG16 attained the highest score across majority of the metrics with 96.97% accuracy, 97.00% F1-score and 99.55% AUC score. ResNet50 and custom CNN ranked second and third respectively across all metrics as summarized in Table 3. The testing accuracy graphs of the three models are presented in Fig. 5. Table 4 present one-way Analysis of Variance (ANOVA) *p*-values testing for testing differences between the three models across all five training rounds. Recall, F1-score and AUC show statistically significant differences among the three models, while precision and specificity do not differ significantly.

**Table 4:** One-way ANOVA of three models deployed for tick identification.

Metrics	*P*-value	Significance ( $\alpha \; =$ 0.05)
Precision	0.3124	Not significant
Recall	0.0013	Significant
F1-score	0.0000	Significant
Specificity	0.3612	Not significant
AUC	0.0000	Significant

### Confusion matrix

#### Ticks *vs* non-ticks

**Figure 6:**
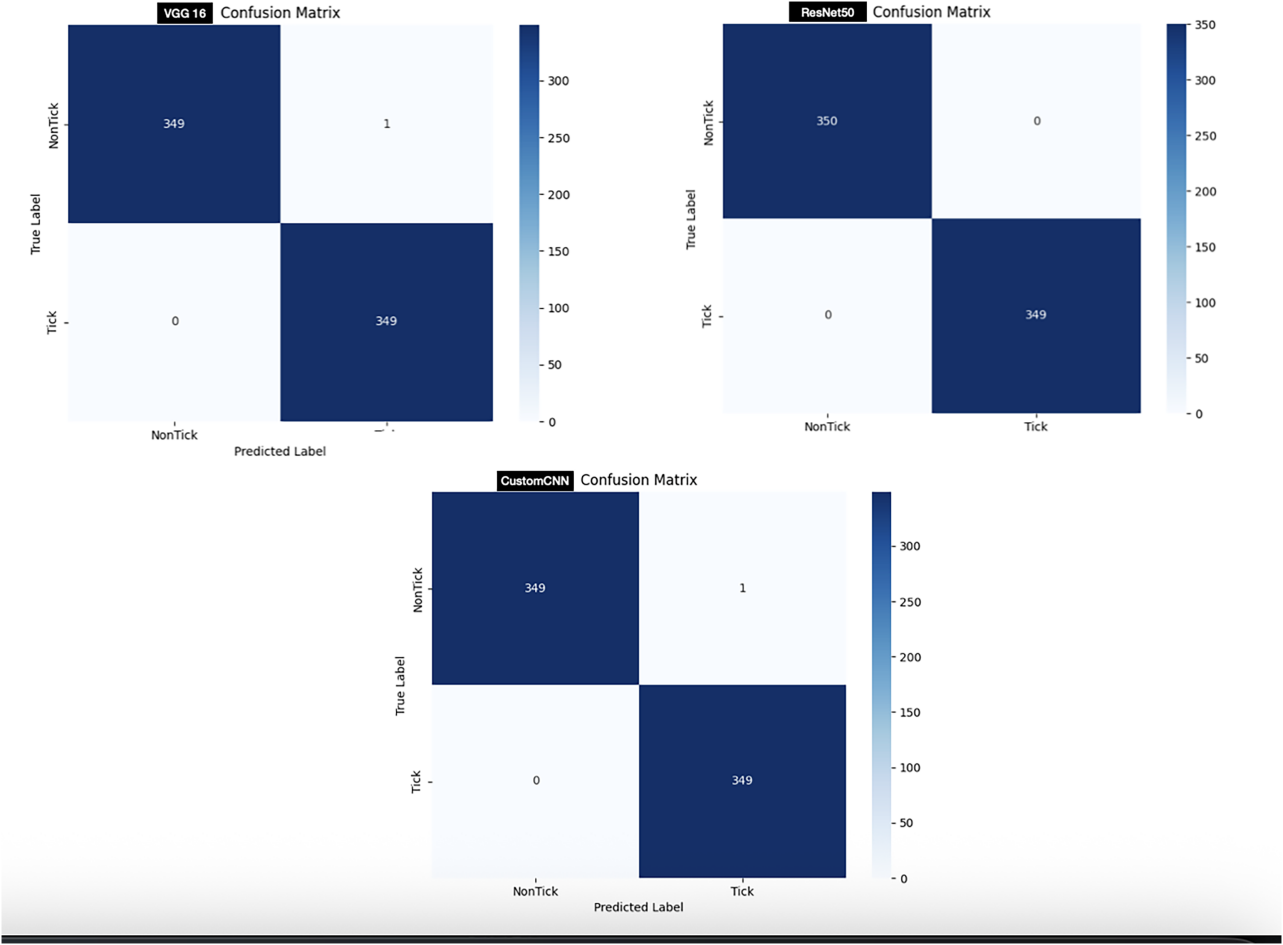
Confusion matrix of ticks *vs* non ticks.

In order to test the models, 700 images (350 images of ticks and 350 images non ticks) are used. As shown in Fig. 6, customized CNN and VGG16 miss-labeled one image of non-tick as tick, while ResNet50 accurately labeled all images, resulting in 0 miss-labelling.

#### Ticks identification

**Figure 7:**
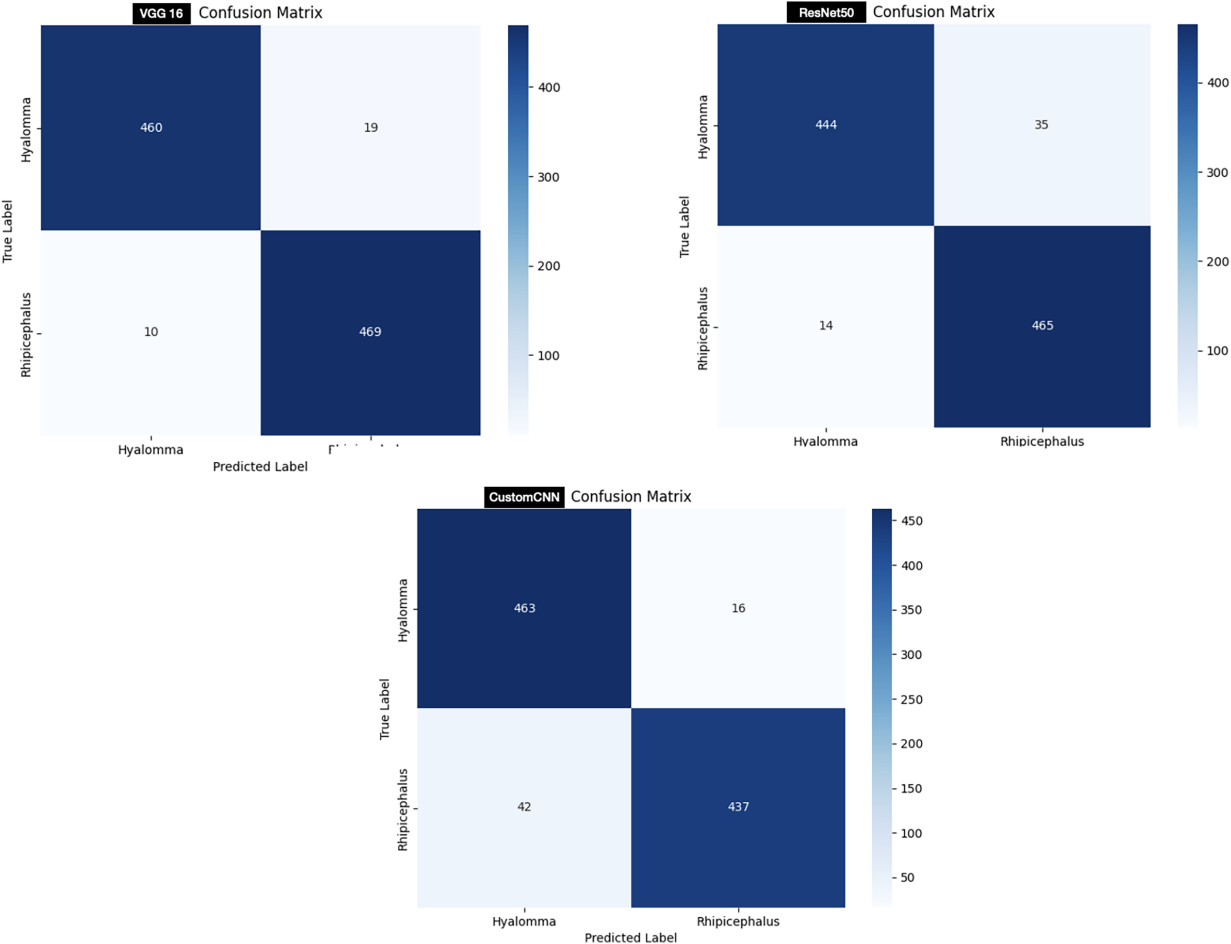
Confusion matrix of ticks identification.

In order to test the models, 958 images (479 Hyalomma and 479 Rhipicephalus images) are used. As shown in Fig. 7, customized CNN miss-labeled 16 images of Hyalomma as Rhipicephalus and 42 images of Rhipicephalus as Hyalomma. VGG16 on the other hand, miss-labeled 19 images of Hyalomma as Rhipicephalus and 10 images of Rhipicephalus as Hyalomma. While ResNet50 miss-labeled 35 images of Hyalomma as Rhipicephalus and 14 images of Rhipicephalus as Hyalomma.

### ROC curve

**Figure 8:**
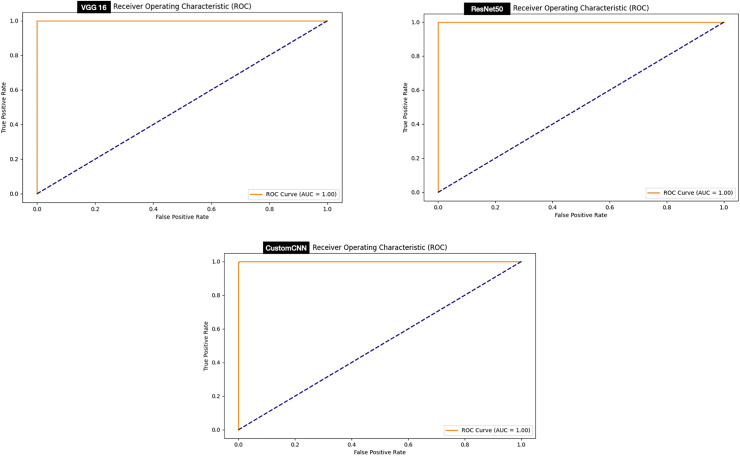
ROC graphs of tick *vs* non ticks.

The ROC curve signifies stable predictions for the model under different thresholds. Evaluation of the ROC curve of the models deployed for the discrimination between ticks and non-ticks has shown that all the models achieved perfect ROC score 100% as summarized in Fig. 8.

**Figure 9:**
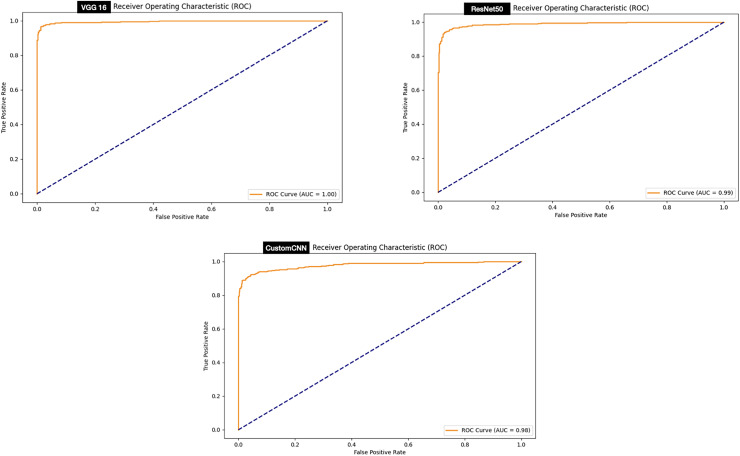
ROC graphs of tick identification.

While, evaluation of the ROC curve of the models deployed for the discrimination between ticks (Hyalomma as Rhipicephalus) has shown that both VGG16 achieved perfect ROC score 100% compare to and ResNet50 (99%) and customized shallow CNN (98%) as summarized in Fig. 9.

### Web development

**Figure 10:**
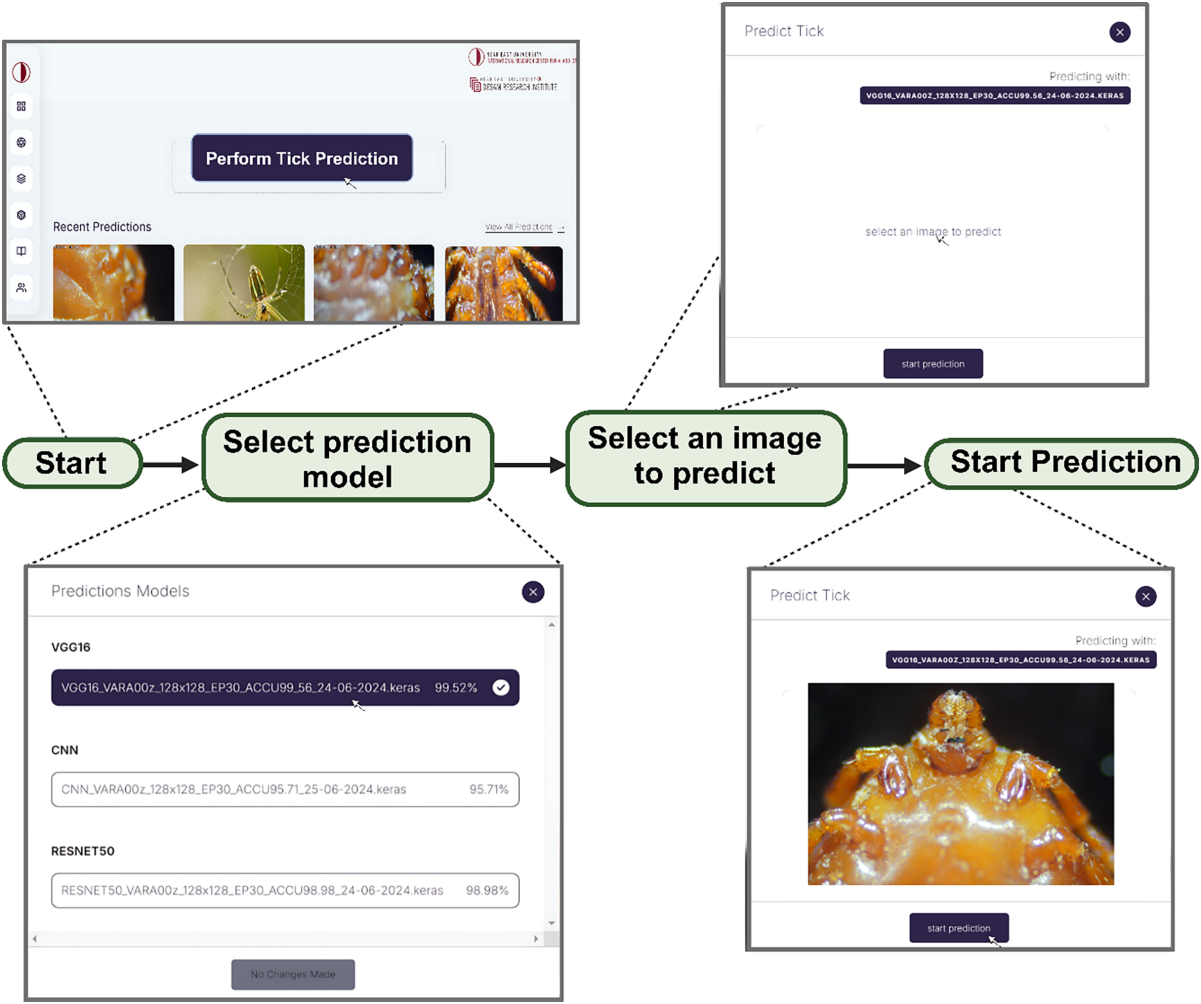
Step by step approach for the detection of tick genera using I-TickNet.

In order to address our second objective, we developed a web app embedded with the three models for the development of DL/IoT based framework that is accessible to the public using the following link: https://tickprediction.aiiot.center/. By using the web app, users can upload images taken by smartphone or cameras and received result in less than a minute. The step-by-step process is illustrated in Fig. 10 and in a video submitted as a Supplemental File. The prediction speed of all the models is presented in Table 5.

**Table 5:** Prediction speed of the deployed models.

Model	Prediction speed for 1 image (ms)	File size (MB)
**Tick identification**		
VGG16	132.55	169.07
ResNet50	75.29	272.6
Custom CNN	32.14	0.29
**Ticks *vs* Non-tick identification**		
VGG16	131.33	169.07
ResNet50	74.18	272.6
CustomCNN	32.57	0.29

## Discussion

This study was conducted to develop a novel web-based application for the accurate classification of tick and non-tick and tick discrimination into Hyalomma and Rhipicephalus *via* implementation of untrained shallow CNN network and two pre-trained models. Moreover, the comparison between untrained and pre-trained models has shown that pre-trained models achieved higher result compare to untrained model.

For the discrimination between ticks and non-ticks, ResNet50 recorded the highest result across all metrics as shown in Table 3. Despite having six layers, custom CNN achieved similar result across all metrics with VGG16. This shows that the three models learn to effectively discriminate between ticks and non-ticks (spiders, beetles, scorpions), due to the clear or subtle differences between the classes. Moreover, this result also coincides with the confusion matrix (Fig. 6), where ResNet50 accurately labelled all the images with single miss-labelling. Furthermore, the overall accuracies of VGG16 and custom CNN reflect in the confusion matrix (Fig. 6), with only 1 miss-labelling.

For the discrimination between Hyalomma and Rhipicephalus, VGG16 recorded the highest result across 6/8 metrics (accuracy, recall, F1-score, AUC, MCC and CK) despite having 16 layers (fewer layers) compared with ResNet50 as shown in Table 3. ResNet50 trailed behind VGG16 based on comparison across metrics. Despite having six layers, custom CNN achieved moderate result across metrics, outperforming both VGG16 and ResNet50 in terms of recall and specificity. This shows that the three models learn to moderately discriminate between Hyalomma and Rhipicephalus. Moreover, similar trend was observed in the confusion matrix (Fig. 7), where VGG16 accurately labelled 939/958 images, resulting in 19 miss-labelling. Furthermore, the overall accuracies of ResNet50 and custom CNN reflect in the confusion matrix (Fig. 7), with 709/758 and 700/758, resulting in 49 and 58 miss-labelling respectively.

Deep learning models, especially untrained model require large amount of training set in order to achieve optimum performance. Despite achieving more than 93% accuracy for tick identification using both pre-trained and untrained models, acquiring more dataset can increase performance. Consequently, increasing diversity in the dataset by including more tick genera is another significant option to improve the model classification efficiency. Another option to maximize training set is true data augmentation. Several data augmentation techniques such as flipping, zooming, shearing, rotation, *etc*., have been reported in the literature and have been shown to improve performance compare to models trained without augmentation. Apart from the dataset, another limitation include the use of two pre-trained models (VGG16 and ResNet50); thus, implementing other pre-trained models such as DenseNet, MobileNet, EfficientNet, Inception, *etc*., can widen the scope of the work. Notwithstanding, some of the existing studies reported the use of ensemble deep learning and machine learning algorithms for classification of medical images. Some of the common classical ML models reported include SVM, K Nearest Neighbor (KNN), Random Forest (RF) *etc*. Therefore, future studies will attempt to integrate traditional ML models.

### Comparison with related work

Here, we conducted a realistic comparison of the proposed approach with existing studies. Thus, we compared the model + classifier that achieved the best result with reported studies in the literature. However, this comparison is not meant to diminish any study or to show that our study is better than the previous studies.

The study proposed by Justen et al. (2021) achieved 87.8% accuracy for three-way classification of ticks using TickIDNet. Despite the training the model with more than 90,000 images, all the three models proposed in this study achieved higher accuracy compare to TickIDNet. The study proposed by Omodior shared a lot of similarities with the current study based on the comparative analysis between customized and pre-trained models. The study achieved 80% mean prediction accuracy using small dataset in comparison with the six-layer base CNN implemented in this study which achieved 93.35%. The result achieved using customized model can be attributed to the large dataset use for training and testing (approximately 9,500).

The study proposed by Luo et al. (2022) achieved an optimum accuracy of 99.5% using Inception V3. The model is trained using augmented image. VGG16 as the best performing model in this study achieved an accuracy of 99.42% and 96.67%. Thus, the InceptionV3 deployed by Luo et al. (2022) slightly ranked higher (99.5%) compare to VGG16. The study proposed by Xu et al. (2021) implemented LeNet model which comprises of five layers. The model is trained using more than 2,000 images which result in 85% validation accuracy, which ranked lower with the base CNN (6-layers) implemented in this study. However, this can be attributed to the extensive pre-processing techniques and larger dataset. The study proposed by Akbarian et al. (2021) achieved 92.04% accuracy using InceptionResNet for the binary classification of black-legged (*Ixodes scapularis*) and non-black-legged ticks. However, all the three models deployed in this study achieved higher accuracy for binary classification compare with InceptionResNet. The summary of comparison with related work is presented in Table 6.

**Table 6:** Comparison with related work.

References	Model	Number of images	Classes	Accuracy
Luo et al. (2022)	Inception V3	12,000	3 (*Dermacentor variabilis*, *Ixodes scapularis* and *Amblyomma americanum*)	99.5%
Xu et al. (2021)	LeNet-SVM	2,000	3 (*Amblyomma americanum*, *Ixodes scapularis* and *Dermacentor variabilis*)	95.69%
Omodior et al. (2021)	ResNet50	2,130	4 (*Amblyomma americanum*, *Ixodes scapularis*, Haemaphysalis sp. and *Dermacentor variabilis*)	80.00%
Justen et al. (2021)	TickIDNet	12,177	3 (*Ixodes scapularis*, *Amblyomma americanum* and *Dermacentor variabilis*)	91.67%
Akbarian et al. (2021)	InceptionResNet	6,294	2 (Blacklegged and non-blacklegged)	92.04%
**This study**	VGG16	9,556	2 (Hyalomma, and Rhipicephalus)	96.97%

### Benefits and risks of automated tick identification

Automated tick identification or discrimination between species using deep learning models like offers significant benefits such as early and rapid detection, improved public health surveillance, reduced costs *etc*. However, despite the advantages of AI-based tick identification, it also poses risks, especially when accuracy and other metrics are below 100%. Some of the risks of this approach include false negative and false positive which can lead to misclassification of harmful tick as harmless. Moreover, overreliance on AI-based approaches can lead to human complacency (users might skip lab confirmation for high-risk bites). Despite the growing literature on the application of AI-based techniques, there are still several issues that need to be addressed which include improving performance of AI-based models on nymphs/engorged ticks and validation in real-world settings. Therefore, future studies can be directed toward the development of hybrid systems that integrates AI-based techniques couple with human experts for validation of high-risk cases, the use of multimodal which support the combination of images and genomic data.

### Clinical or practical implication

As a result of the increasing number of Crimean-Congo hemorrhagic fever (CCHF) and Mediterranean spotted fever caused by Hyalomma and Rhipicephalus, developing an AI/IoT-based identification system for tick genera can offer significant clinical and public health implications. AI/IoT-based identification system for tick genera can enhanced disease surveillance and outbreak prevention through early detection of pathogenic vectors, mapping high risk regions. Moreover, this platform supports faster and accurate identification of the two common tick genera by reducing misidentification. AI/IoT-powered platforms such as I-TickNet can be integrated into laboratory settings in order to serve as a confirmatory system as well as in agriculture and veterinary for livestock protection (Hyalomma and Rhipicephalus infest livestock) and wildlife reservoir tracking.

### Limitations and future work

While genus-level identification is a valuable first step and provides some useful information, however, it is insufficient on its own to plan effective and targeted control measures. Genus-level identification is helpful as it provides information of the genus of interest (*i.e*., Ixodes, Amblyomma, Dermacentor, Rhipicephalus *etc*.), general life cycle, preferred hosts (*i.e*., Ixodes prefer mammals, birds, and reptiles, while Amblyomma often targets large mammals), seasonal activity, associated diseases (*i.e*., Ixodes are associated with Lyme disease, Amblyomma with Ehrlichiosis and Heartland virus, Dermacentor with Rocky Mountain Spotted Fever and tularaemia). Therefore, species-level identification is more critical for effective control, as it enables public health officials and pest control experts to target specific behavior, life cycle, and ecology of the pest tick, thereby, maximizing the impact of control interventions while minimizing environmental disruption and cost.

In order to directly address the need for broader validation and ensure generalizability, our future work will include a clear, multi-faceted plan:

- We are planning to seek collaborations with research institutions in different climatic and ecological regions (*e.g*., Southern Europe, North America, Asia) to test I-TickNet on tick specimens native to those areas. This will test its performance against different phenotypic variations within the same genera.
- The most immediate next step is to expand the model’s capability beyond Hyalomma and Rhipicephalus. We plan to include other medically important genera like Ixodes, Dermacentor, and Amblyomma.
- We plan to deploy I-TickNet in a controlled field setting with veterinary clinics or public health units in Northern Cyprus. This will allow us to gather data on its performance with real-time, user-submitted images of varying quality, which is the ultimate test for a field application.

## Conclusions

Ticks and tick-borne diseases are categorized among diseases with global concern by numerous international and health organizations such as the United Nations (UN), European Union (EU), World Health Organization (WHO), Centers for Disease Control and Prevention (CDC), *etc*. In the last decades, incidence of vector-borne diseases has increased significantly, and therefore, there is a need for accurate identification of vectors, including ticks. Normally, the identification and classification of ticks are conducted by an expert entomologist. However, an easier, faster, more accurate and more sensitive method is crucial to take control of vectors. Therefore, there is a need to develop an accurate and real-time framework that can easily be used by the non-professionals to differentiate various tick genera. To address these issues, scientists adopted DL-based technique as a subsidiary of ML and AI. DL models have been implemented in several fields for image classification and detection, resulting excellent results. In congruence with previous studies, in this study, we have implemented an untrained CNN model and two pre-trained models for binary classification of Hyalomma and Rhipicephalus cases. The models are trained and tested using 9,556 images taken from 35 different ticks after the completion of their identification process by acarologists.

The performance evaluation of the models has indicated that all the three models achieved an accuracy of more than 90%. Comparative analysis between performances of the models deployed for discrimination of ticks and non-ticks has shown that ResNet50 achieved an optimum 100% across all metrics. While, for discrimination between Hyalomma and Rhipicephalus, VGG16 achieved the best result with 96.97% accuracy. Considering the fact that there are around 900 tick species, acquiring more dataset of both transmissive and non-transmissive ticks can improve the performance of the model. Moreover, the study can be modified by exploring other DL models such as MobileNet, ResNet101, ResNet152, DenseNet and its variants, EfficientNet and its variants, inception *etc*. Moreover, future studies will also explore ensemble learning by combining both DL models and ML classifiers such as SVM, Decision Tree (DT), KNN *etc*. In addition, future study will also focus on developing AI-based tick vector tracking system that support identification of ticks and surveillances.

The data altogether indicated that the VGG16 model is the most suitable model to be used for deep learning of the ticks. The proposed model demonstrated a lower rate of errors and had a higher accuracy of identification of tick genera making it superior over other models tested. This study demonstrated an easier and faster approach of tick identification which might assist in vector tracking programs to take the control of any possible vector borne outbreaks in the future.

## Supplemental Information

10.7717/peerj-cs.3291/supp-1 Supplemental Information 1 README for training code.

## Additional Information and Declarations

### Data Availability

The following information was supplied regarding data availability:

The data is available at Zenodo:

- Ame, I., & Al-Turjman, F. (2024). Tick Dataset for ITICK version A [Data set]. Zenodo. https://doi.org/10.5281/zenodo.14353933.

- Ame, I. (2024). Training Code For ITICK Models version A. Zenodo. https://doi.org/10.5281/zenodo.14770030.

## References

[ref-2] Akbarian S, Nelder MP, Russell CB, Cawston T, Moreno L, Patel SN, Allen VG, Dolatabadi E (2021). A computer vision approach to identifying ticks related to Lyme disease. IEEE Journal of Translational Engineering in Health and Medicine.

[ref-3] Barker SC, Murrell A (2004). Systematics and evolution of ticks with a list of valid genus and genera names. Parasitology.

[ref-4] Dantas-Torres F (2018). Species concepts: what about ticks?. Trends in Parasitology.

[ref-5] Estrada-Peña A (2015). Ticks as vectors: taxonomy, biology and ecology. Revue Scientifique et Technique.

[ref-6] Geevarghese G, Mishra AC (2011). Haemaphysalis ticks of India.

[ref-7] He K, Zhang X, Ren S, Sun J (2016). Deep residual learning for image recognition.

[ref-8] Ibrahim AU (2023). Deep learning-based tick image classification using CNN models. Master’s thesis, Near East University, Mersin, Turkey.

[ref-9] Ibrahim AU, Engo GM, Ame I, Nwekwo CW, Al-Turjman F (2025a). I-BrainNet: deep learning and internet of things (DL/IoT)—based framework for the classification of brain tumor. Journal of Imaging Informatics in Medicine.

[ref-10] Ibrahim AU, Kibarer AG, Al-Turjman F, Kaba S (2023). Large-scaled detection of COVID-19 from X-ray using transfer learning. International Journal of Imaging Systems and Technology.

[ref-11] Ibrahim AU, Nwaneri IO, Vubangsi M, Al-Turjman F (2025b). I-Brainer: artificial intelligence/internet of things (AI/IoT)-powered detection of brain cancer.

[ref-12] Ibrahim AU, Ozsoz M, Serte S, Al-Turjman F, Yakoi PS (2024). Pneumonia classification using deep learning from chest X-ray images during COVID-19. Cognitive Computation.

[ref-13] Irkham I, Ibrahim AU, Nwekwo CW, Al-Turjman F, Hartati YW (2022). Current technologies for detection of COVID-19: biosensors, artificial intelligence and internet of medical things (IOMT). Sensors.

[ref-14] Justen L, Carlsmith D, Paskewitz SM, Bartholomay LC, Bron GM (2021). Identification of public submitted tick images: a neural network approach. PLOS ONE.

[ref-15] Kim HE, Cosa-Linan A, Santhanam N, Jannesari M, Maros ME, Ganslandt T (2022). Transfer learning for medical image classification: a literature review. BMC Medical Imaging.

[ref-16] Luo CY, Pearson P, Xu G, Rich SM (2022). A computer vision-based approach for tick identification using deep learning models. Insects.

[ref-17] Mascarenhas S, Agarwal M (2021). A comparison between VGG16, VGG19 and ResNet50 architecture frameworks for image classification.

[ref-18] Omodior O, Saeedpour-Parizi MR, Rahman MK, Azad A, Clay K (2021). Using convolutional neural networks for tick image recognition—a preliminary exploration. Experimental and Applied Acarology.

[ref-19] Ruenchit P (2021). State-of-the-art techniques for diagnosis of medical parasites and arthropods. Diagnostics.

[ref-20] Salehi AW, Khan S, Gupta G, Alabduallah BI, Almjally A, Alsolai H, Siddiqui T, Mellit A (2023). A study of CNN and transfer learning in medical imaging: advantages, challenges, future scope. Sustainability.

[ref-21] Sengupta A, Ye Y, Wang R, Liu C, Roy K (2019). Going deeper in spiking neural networks: VGG and residual architectures. Frontiers in Neuroscience.

[ref-22] Shah T, Li Q, Wang B, Baloch Z, Xia X (2023). Geographical distribution and pathogenesis of ticks and tick-borne viral diseases. Frontiers in Microbiology.

[ref-23] Simonyan K, Vedaldi A, Zisserman A (2013). Deep inside convolutional networks: visualising image classification models and saliency maps.

[ref-24] Tamuly S, Jyotsna C, Amudha J (2020). Deep learning model for image classification.

[ref-25] Targ S, Almeida D, Lyman K (2016). ResNet in ResNet: generalizing residual architectures.

[ref-26] Tsatsaris A, Chochlakis D, Papadopoulos B, Petsa A, Georgalis L, Angelakis E, Ioannou I, Tselentis Y, Psaroulaki A (2016). Species composition, distribution, ecological preference and host association of ticks in Cyprus. Experimental and Applied Acarology.

[ref-27] Vieira Lista MC, Belhassen-García M, Vicente Santiago MB, Sánchez-Montejo J, Pedroza Pérez C, Monsalve Arteaga LC, Herrador Z, del Álamo-Sanz R, Benito A, Soto López JD, Muro A (2022). Identification and distribution of human-biting ticks in Northwestern Spain. Insects.

[ref-28] Wang P, Fan E, Wang P (2021). Comparative analysis of image classification algorithms based on traditional machine learning and deep learning. Pattern Recognition Letters.

[ref-29] Xu Z, Ding X, Yin K, Li Z, Smyth JA, Sims MB, McGinnis HA, Liu C (2021). Tickphone App: a smartphone application for rapid tick identification using deep learning. Applied Sciences.

[ref-30] Zhuang F, Qi Z, Duan K, Xi D, Zhu Y, Zhu H, Xiong H, He Q (2020). A comprehensive survey on transfer learning. Proceedings of the IEEE.

